# Prevalence of left ventricular diastolic dysfunction in European populations based on cross-validated diagnostic thresholds

**DOI:** 10.1186/1476-7120-10-10

**Published:** 2012-03-19

**Authors:** Malgorzata Kloch-Badelek, Tatiana Kuznetsova, Wojciech Sakiewicz, Valérie Tikhonoff, Andrew Ryabikov, Arantxa González, Begoña López, Lutgarde Thijs, Yu Jin, Sofia Malyutina, Katarzyna Stolarz-Skrzypek, Edoardo Casiglia, Javier Díez, Krzysztof Narkiewicz, Kalina Kawecka-Jaszcz, Jan A Staessen

**Affiliations:** 1 The First Department of Cardiology and Hypertension, Jagiellonian University, Kraków, Poland; 2 The Studies Coordinating Centre, Division of Hypertension and Cardiovascular Rehabilitation, Department of Cardiovascular Diseases, University of Leuven, Leuven, Belgium; 3 Hypertension Unit, Department of Hypertension and Diabetology, Medical University of Gdańsk, Gdańsk, Poland; 4 The Department of Clinical and Experimental Medicine, University of Padova, Padova, Italy; 5 The Institute of Internal Medicine, Novosibirsk, Russian Federation; 6 Division of Cardiovascular Sciences, Centre for Applied Medical Research, Pamplona, Spain; 7 Department of Epidemiology, Maastricht University, Maastricht, The Netherlands; 8 Division of Hypertension and Cardiac Rehabilitation, Department of Cardiovascular Diseases, University of Leuven, Campus Sint Rafaël, Kapucijnenvoer 35, Block D, Box 7001, B-3000 Leuven, Belgium

**Keywords:** Epidemiology, Echocardiography, Tissue Doppler Imaging, Diastole

## Abstract

**Background:**

Different diagnostic criteria limit comparisons between populations in the prevalence of diastolic left ventricular (LV) dysfunction. We aimed to compare across populations age-specific echocardiographic criteria for diastolic LV dysfunction as well as their correlates and prevalence.

**Methods:**

We measured the E and A peaks of transmitral blood flow by pulsed wave Doppler and the e' and a' peaks of mitral annular velocities by tissue Doppler imaging (TDI) in 2 cohorts randomly recruited in Belgium (*n *= 782; 51.4% women; mean age, 51.1 years) and in Italy, Poland and Russia (*n *= 476; 55.7%; 44.5 years).

**Results:**

In stepwise regression, the multivariable-adjusted correlates of the transmitral and TDI diastolic indexes were similar in the 2 cohorts and included sex, age, body mass index, blood pressure and heart rate. Similarly, cut-off limits for the E/A ratio (2.5th percentile) and E/e' ratio (97.5th percentile) in 338 and 185 reference subjects free from cardiovascular risk factors respectively selected from both cohorts were consistent within 0.02 and 0.26 units (median across 5 age groups). The rounded 2.5th percentile of the E/A ratio decreased by ~0.10 per age decade in these apparently healthy subjects. The reference subsample provided age-specific cut-off limits for normal E/A and E/e' ratios. In the 2 cohorts combined, diastolic dysfunction groups 1 (impaired relaxation), 2 (possible elevated LV filling pressure) and 3 (elevated E/e' and abnormally low E/A) encompassed 114 (9.1%), 135 (10.7%), and 40 (3.2%) subjects, respectively.

**Conclusions:**

The age-specific criteria for diastolic LV dysfunction were highly consistent across the study populations with an age-standardized prevalence of 22.4% vs. 25.1%.

## Background

Cardiovascular risk factors underlie the first stage of diastolic heart failure (HF). This stage evolves into asymptomatic left ventricular diastolic dysfunction (LVDD) characterized by impaired relaxation and increased left ventricular (LV) stiffness, and finally progresses to clinically overt diastolic HF [1]. Recently published community-based studies making use of conventional and tissue Doppler echocardiographic imaging (TDI) revealed a high prevalence of LVDD, ranging from 11.1% up to 34.7% [2-8]. In the Flemish Study on Environment, Genes and Health Outcomes (FLEMENGHO), the frequency was 27.3% [9]. One issue making the interpretation of the aforementioned reports difficult is that the prevalence of LVDD cannot be easily compared, partially because of differing diagnostic criteria and/or divergent distributions of cardiovascular risk factors in the sampled populations (for review see Additional file 1: Tables S1 and S2). In the FLEMENGHO study, [9] we derived age-specific criteria for the classification of LVDD based on a reference sample. Since the publication of our previous report, [9] echocardiographic examinations continued in the FLEMENGHO cohort and started in four centers taking part in the European Project on Genes in Hypertension (EPOGH). In the present study, we compared age-specific echocardiographic criteria for LVDD across populations as well as the correlates and prevalence of this condition. By showing consistency across populations, we aimed to propose a diagnostic classification that might be useful in clinical practice.

## Methods

### Study participants

From August 1985 until December 2005, we randomly recruited a family-based population sample from a geographically defined area in northern Belgium as described elsewhere [9]. EPOGH recruited participants from 1999 until 2001. The EPOGH investigators were trained at the Studies Coordinating Centre, and applied the same protocol, questionnaires and follow-up procedures, as used in FLEMENGHO. The FLEMENGHO and EPOGH studies received ethical approval. The initial response rate at enrolment was 61.3% [10,11]. All subjects provided informed consent in writing.

The FLEMENGHO and EPOGH participants remained in follow-up. Five centers opted to perform echocardiographic phenotyping and to assess LV function by using the new TDI indexes along with classical pulsed wave Doppler velocities of blood flow. Our current study population includes 1287 subjects, who were examined from June 2005 until September 2009. We excluded 29 subjects from analysis, because of atrial fibrillation (*n *= 12), a pacemaker (*n *= 2), or because LVDD could not be reliably determined (*n *= 15). Thus, the current analysis included 1258 participants: 782 FLEMENGHO participants (Noorderkempen, Belgium) and 476 EPOGH subjects from Gdańsk (*n *= 108) and Kraków (*n *= 124), Poland, Mirano, Italy (n = 106), and Novosibirsk, Russia (*n *= 138).

### Echocardiography

In each center one experienced physician did the ultrasound examination, using a Vivid 7 Pro (GE Vingmed, Horten, Norway), interfaced with a 2.5- to 3.5-MHz phased-array probe, according to a standardized protocol described in detail in previous publications [9]. With the subjects in partial left decubitus and breathing normally, the observer obtained images, together with a simultaneous ECG signal, along the parasternal long and short axes and from the apical 4-, 2-chamber and long-axis views. All recordings included at least 5 cardiac cycles and were digitally stored for off-line analysis.

One experienced observer (TK) analyzed the digitally stored images, using the EchoPac software, version 4.0.4 (GE Vingmed), averaging three cardiac cycles. The LV internal diameter and interventricular septal and posterior wall thickness were measured at end-diastole from the 2-dimensionally guided M-mode tracings according to the recommendations [12]. When optimal orientation of M-mode ultrasound beam could not be obtained, the reader performed linear measurements on correctly oriented two-dimensional images. End-diastolic LV dimensions were used to calculate LV mass by an anatomically validated formula. LV hypertrophy was a left ventricular mass index (LVMI) of 125 g/m^2 ^in men and 110 g/m^2 ^in women or more. We calculated LV ejection fraction (EF) from LV end-systolic and end-diastolic volumes measured from the apical 4- and 2-chambers views, using the standard biplane Simpson's method. We measured left atrial (LA) dimensions in 3 orthogonal planes: the parasternal long, lateral, and supero-inferior axes [13]. LA volume (LAVI) was calculated using the prolate-elipsoid method [13] and was indexed to body surface area.

From the transmitral flow signal, we measured peak early (E) and late (A) diastolic velocities, the E/A ratio and A flow duration. The duration of PV reversal flow during atrial systole (AR) was measured from the PV flow signal. From the pulsed wave TDI recordings, we measured the early (e') and late (a') peak diastolic velocities of the mitral annulus displacement, and the e'/a' ratio at the 4 acquisition sites (septal, lateral, inferior, and posterior). We calculated the E/e' ratio by dividing transmitral E peak by e' averaged from the 4 acquisition sites. As reported previously, [9] the inter-observer intra-session reproducibility across the four sampling sites ranged from 4.48% to 5.34% for e' velocities and from 3.96% to 4.52% for a' velocities.

### Other measurements

We administered a standardized questionnaire to collect detailed information on each subject's medical history, smoking and drinking habits, and intake of medications. The conventional blood pressure was the average of five consecutive auscultatory readings obtained with the subject in the seated position. Hypertension was a blood pressure of at least 140 mmHg systolic or 90 mmHg diastolic or the use of antihypertensive drugs. Body mass index (BMI) was weight in kilograms divided by the square of height in meters. Obesity was a BMI of 30 kg/m^2 ^or higher. Central obesity was a waist circumference of at least 102 cm in men or 88 cm in women. Diabetes was a fasting blood glucose of 7.0 mmol/L or higher or the use of antidiabetic agents.

NT-proBNP in the FLEMENGHO study was measured in plasma by a competitive enzymatic immunoassay for research use (Biomedica Gruppe, Vienna, Austria) [14]. NT-proBNP in the EPOGH study was determined in serum by an electrochemiluminescence immunoassay Elecsys 2010 (Roche Diagnostics, Indianapolis, USA) [14].

To generate a reference sample, we excluded participants, if one or more of the following conditions were present: hypertension (FLEMENGHO/EPOGH, n = 323/211), diabetes (n = 29/25), obesity (n = 143/134), central obesity (n = 216/144), LV hypertrophy (n = 84/36), renal failure (n = 4/4) or cardiac diseases (valvular abnormalities, n = 31/8; myocardial infarction and/or coronary revascularization, n = 24/6). The number of subjects in the reference group was 338 in FLEMENGHO, 185 in EPOGH, and 523 in total.

### Statistical methods

For database management and statistical analysis, we used SAS software, version 9.1.3 (SAS Institute, Cary, NC). We compared means and proportions by means of a large sample z-test and the **χ**^2^-test, respectively. We performed single and stepwise linear regression to identify correlates of the Doppler indexes as measured on a continuous scale. We set the *P*-values for variables to enter and to stay in the regression models at 0.05. To obtain 95% confidence intervals of the percentile values of the E/A distributions, we computed the bootstrap distribution [15] of the thresholds by randomly resampling the study population 1000 times with replacement, using the PROC SURVEYSELECT procedure, as implemented in the SAS software. Because NT-proBNP was measured by different methods in FLEMENGHO and EPOGH, we rescaled the values by computing population-specific z-scores, which reflect the deviation of each individual measurement from the population mean.

## Results and discussion

### Characteristics of participants

The 1258 participants included 666 (52.9%) women, and 534 (42.5%) hypertensive patients of whom 325 (26.0%) were on antihypertensive drug treatment. Mean age (± SD) was 48.5 ± 15.7 years. Tables 1 and 2 list the clinical and echocardiographic characteristics of the FLEMENGHO and EPOGH participants in the entire study population and in the reference groups. The participants were older in FLEMENGHO than in EPOGH. LAVI, LV wall thickness, LVMI and EF (Table 2) were greater in FLEMENGHO than in EPOGH participants. Only 11 subjects (0.9%) had an EF less than 50%. The FLEMENGHO participants also had lower E/A and e'/a' ratios (Table 2). Additional file 1: Tables S3 and S4 show the clinical and echocardiographic characteristics of the EPOGH participants by center.

**Table 1:** Characteristics of participants

	Entire population		Reference group	
Characteristic	FLEMENGHO (n = 782)	EPOGH (n = 476)	FLEMENGHO (n = 338)	EPOGH (n = 185)
Anthropometrics				
Women, n (%)	402 (51.4)	265 (55.7)	172 (50.9)	110 (59.8)
Age, y	51.1 ± 15.4	44.5 ± 15.1‡	42.5 ± 13.1	34.6 ± 11.7‡
Height, cm	168.7 ± 9.46	168.0 ± 9.0	170.4 ± 9.1	168.7 ± 8.76*
Weight, kg	75.4 ± 14.4	76.3 ± 16.4	69.7 ± 11.4	66.1 ± 11.8‡
Body mass index, kg/m^2^	26.5 ± 4.31	27.0 ± 5.21	24.0 ± 2.76	23.1 ± 3.2†
Waist circumference, cm	89.9 ± 12.3	89.6 ± 14.1	82.5 ± 9.24	79.2 ± 9.9‡
Systolic pressure, mm Hg	129.5 ± 17.6	130.5 ± 19.9	118.3 ± 9.5	117.1 ± 11.5
Diastolic pressure, mm Hg	79.6 ± 9.46	81.3 ± 12.4*	75.2 ± 7.32	74.1 ± 7.9
Heart rate, beats/minute	60.8 ± 9.63	67.0 ± 10.6‡	60.3 ± 8.8	66.3 ± 9.5‡
Questionnaire data				
Current smoking, n (%)	167 (21.4)	102 (21.9)	98 (29.0)	40 (21.9)
Drinking alcohol, n (%)	312 (39.9)	144 (30.2)‡	157 (46.4)	43 (23.2)‡
Hypertensive, n (%)	323 (41.3)	211 (44.3)	...	...
Treated for hypertension, n (%)	198 (25.32)	127 (27.2)	...	...
Diabetes, n (%)	29 (3.71)	25 (5.25)	...	...

Values are mean (± SD), or number of subjects (%). *P ≤ 0.05; †*P *≤ 0.01; ‡*P *≤ 0.001

**Table 2:** Echocardiographic characteristics of participants

	Entire population		Reference group	
Characteristics	FLEMENGHO (n = 782)	EPOGH (n = 476)	FLEMENGHO (n = 338)	EPOGH (n = 185)
Conventional echocardiography				
Left atrium volume index, ml/m^2^	22.9 ± 6.3	21.0 ± 5.6‡	20.0 ± 4.3	18.2 ± 3.8‡
LV internal diameter, cm	5.04 ± 0.49	5.00 ± 0.43	4.99 ± 0.43	4.88 ± 0.40†
Interventricular septum, cm	0.98 ± 0.17	0.95 ± 0.16‡	0.90 ± 0.13	0.85 ± 0.12‡
Posterior wall, cm	0.89 ± 0.14	0.87 ± 0.13* 0.82 ± 0.12		0.79 ± 0.11*
LV mass index, g/m^2^	92.5 ± 21.9	87.9 ± 20.4‡	82.9 ± 15.4	77.9 ± 15.5‡
Ejection fraction,%	68.2 ± 7.4	62.6 ± 5.9‡	67.1 ± 6.3	61.3 ± 5.5‡
Doppler data				
E peak, cm/s	75.4 ± 15.9	71.6 ± 15.3‡	79.7 ± 14.7	76.1 ± 14.6†
A peak, cm/s	65.0 ± 17.5	58.6 ± 16.8‡	55.6 ± 12.8	48.4 ± 11.6‡
E/A ratio	1.26 ± 0.48	1.33 ± 0.52†	1.52 ± 0.49	1.66 ± 0.50†
e' peak#, cm/s	11.4 ± 3.6	11.2 ± 3.6	13.8 ± 3.1	13.7 ± 2.6
a' peak#, cm/s	10.1 ± 2.1	9.23 ± 2.1‡	9.37 ± 2.1	8.19 ± 2.0‡
e'/a' ratio#	1.24 ± 0.64	1.36 ± 0.70†	1.63 ± 0.68	1.84 ± 0.66‡
E/e' ratio	7.08 ± 2.2	6.88 ± 2.2	5.93 ± 1.2	5.65 ± 1.1†

Values are mean (± SD). # Averaged of septum, lateral, inferior and posterior mitral annulus sites. * *P *≤ 0.05; †*P*≤ 0.01; ‡*P *≤ 0.001.

### Determinants of transmitral and TDI velocity ratios

In FLEMENGHO and EPOGH subjects, the E/A and e'/a' ratios significantly and independently decreased with age, BMI, heart rate and diastolic blood pressure (Table 3). Furthermore, the e'/a' ratio decreased with higher LVMI in the EPOGH participants. In both cohorts, the transmitral and mitral annular velocities ratios increased with systolic blood pressure. The E/e' ratio increased with female sex, age, BMI, systolic blood pressure, and LVMI (Table 3). The E/e' ratio decreased with higher diastolic blood pressure and LV length in both studies and with heart rate in FLEMENGHO participants. There were no differences in the partial regression coefficients between both studies (*P *≥ 0.10).

**Table 3:** Correlates of the E/A, e'/a' and E/e' ratios in stepwise regression by cohort

Parameter	Transmitral E/A		Averaged TDI e'/a'		E/e'	
	FLEMENGHO	EPOGH	FLEMENGHO	EPOGH	FLEMENGHO	EPOGH
R^2 ^(%)	67.5	65.9	73.6	71.2	49.6	46.2
Adjusted R^2 ^(%)	67.3	65.4	73.4	70.6	49.0	45.1
Partial regression coefficients						
Female (0,1)						
ß ± SE			0.064 ± 0.025*	0.084 ± 0.039*	0.62 ± 0.15‡	0.37 ± 0.19*
Partial r^2 ^(%)			0.22	0.29	1.4	0.44
Age (+10 years)						
ß ± SE	-0.23 ± 0.008‡	-0.24 ± 0.012‡	-0.32 ± 0.009‡	-0.28 ± 0.015‡	0.38 ± 0.048‡	0.50 ± 0.068‡
Partial r^2 ^(%)	53.6	51.4	61.1	54.4	33.6	35.4
BMI (+1 kg/m^2^)						
ß ± SE	-0.017 ± 0.003‡	-0.011 ± 0.00‡	-0.031 ± 0.003‡	-0.026 ± 0.004‡	0.093 ± 0.015‡	0.078 ± 0.017‡
Partial r^2 ^(%)	2.5	0.78	6.8	6.5	3.1	2.3
HR (+10 beats/minute)						
ß ± SE	-0.13 ± 0.011‡	-0.13 ± 0.014‡	-0.11 ± 0.013‡	-0.13 ± 0.017‡	-0.16 ± 0.063†	
Partial r^2 ^(%)	9.5	8.6	3.2	4.1	0.46	
SBP (+10 mmHg)						
ß ± SE	0.025 ± 0.008†	0.030 ± 0.012*	0.044 ± 0.010‡		0.36 ± 0.045‡	0.36 ± 0.068‡
Partial r^2 ^(%)	0.43	0.43	0.7		5.1	4.2
DBP (+10 mmHg)						
ß ± SE	-0.075 ± 0.013‡	-0.079 ± 0.019‡	-0.12 ± 0.016‡	-0.071 ± 0.017‡	-0.21 ± 0.076†	-0.27 ± 0.10†
Partial r^2 ^(%)	0.97	1.5	1.7	1.7	0.70	0.82
LVMI (+10 g/m^2^)						
ß ± SE				-0.025 ± 0.011*	0.16 ± 0.032‡	0.13 ± 0.049†
Partial r^2 ^(%)				0.67	1.4	0.49
LV length (+1 cm)						
ß ± SE					-0.53 ± 0.10‡	-0.45 ± 0.13‡
Partial r^2 ^(%)					3.8	1.8

Values are mutually adjusted partial regression coefficients ± SE. BMI, body mass index; HR, heart rate; SBP, systolic blood pressure; DBP, diastolic blood pressure; LVMI, left ventricle mass index. Significance of the partial regression coefficient: * *P *< 0.05, †*P *< 0.01, and ‡*P *< 0.001. There were no differences between the partial regression coefficients in two studies (*P *≥ 0.10)

### Transmitral and TDI Doppler indexes in the reference groups

Additional file 1: Tables S5, S6 and S7 show the age-specific percentiles of the E/A, e'/a' and E/e' ratios, and LAVI in reference participants free from any cardiovascular disease or risk factors selected from the entire study population, FLEMENGHO and EPOGH, respectively. There was a significant decline in the E/A and e'/a' ratio with age even in the reference group (*P *< 0.0001), because of significant decreases in E and e' velocities and increases in A and a' velocities (Additional file 1: Table S8). In the reference group (Additional file 1: Table S5), the E/e' ratio increased with age (*P *< 0.0001). However, the 97.5 percentile of the E/e' ratio in the entire reference group (8.60) approximated to the 8.5 cut-off limit for the normal filling pressure as observed in invasive studies [16]. In the 523 participants from the reference group, the 97.5 percentile of LAVI was 28.3 ml/m^2^.

Table 4 shows the 2.5 and 97.5 age-group specific percentiles for the transmitral E/A ratio and the 97.5 percentiles for E/e' in all reference subjects and in the FLEMEMGHO and EPOGH reference groups. Using the bootstrap approach, we calculated the confidence intervals of the 2.5 or 97.5 percentiles in the combined reference group by age categories. Next, we rounded these 2.5 and 97.5 age-specific percentiles to the closest integer value. All of these rounded thresholds fell within the 95% confidence boundaries for the 2.5 and 97.5 age-specific percentiles of the E/A ratio and the 97.5 percentiles of the E/e' ratio in the reference group (Table 4).

**Table 4:** Age-specific percentiles for the E/A and E/e' ratio in the reference groups

Age group (years)	FLEMENGHO (n = 338)	EPOGH (n = 185)	All subjects* (n = 523)	95% CI for the age-specific percentiles	Rounded limits
*Transmitral E/A*	**2.5 percentile**				
< 30	1.23	1.23	1.23	1.17 to 1.31	1.20
30-39	1.15	0.98	1.04	0.98 to 1.15	1.00
40-49	0.88	0.95	0.91	0.87 to 0.95	0.90
50-59	0.76	0.78	0.78	0.75 to 0.84	0.80
≥ 60	0.67	0.67	0.67	0.66 to 0.74	0.70
	**97.5 percentile**				
< 30	3.22	3.25	3.18	2.87 to 3.25	3.20
30-39	2.82	2.55	2.62	2.39 to 2.93	2.60
40-49	2.36	2.05	2.22	2.04 to 2.51	2.20
50-59	1.61	1.72	1.63	1.55 to 1.72	1.60
≥ 60	1.55	1.28	1.48	1.28 to 1.55	1.50
*E/e'*	**97.5 percentile**				
< 30	7.66	7.80	7.66	6.98 to 8.17	8.00
30-39	7.47	8.24	7.47	7.04 to 8.56	8.00
40-49	8.85	8.15	8.52	7.64 to 8.85	8.50
50-59	8.84	8.83	8.84	8.60 to 9.62	9.00
≥ 60	8.99	8.73	8.99	8.73 to 9.00	9.00

* The average of the 2.5 and 97.5 percentiles obtained in the FLEMENGHO and EPOGH cohorts

### Prevalence of LVDD

We combined the mitral inflow and TDI velocities to classify the stages of LVDD. The first group included subjects with an abnormally low age-specific transmitral E/A ratio indicative of impaired relaxation (less than rounded 2.5 percentile; Table 4), but without evidence of increased LV filling pressures (E/e' 8.5). The second group had mildly-to-moderately elevated LV filling pressure (E/e' > 8.5), and E/A ratio within the normal age-specific range (the rounded 2.5 to 97.5 percentile; Table 4). We used the differences in durations between the mitral A flow and the reverse PV flow during atrial systole (Ad < ARd + 10) and/or LAVI (28 ml/m^2^) *to confirm *possible elevation of the LV filling pressures in group 2. Group 3 had combined LVDD with an elevated E/e' ratio and an abnormally low age-specific E/A ratio. LVDD groups 1, 2 and 3 included 114 (9.1%), 135 (10.7%), and 40 (3.2%) subjects, respectively. Table 5 presents the prevalence of LVDD by study population, center, and age group respectively. With standardization to mean age, there were no differences in the prevalence of LVDD between the FLEMENGHO and EPOGH cohorts (22.4% vs. 25.1%; *P *= 0.09). However, in the EPOGH participants, the age-standardized prevalence of LVDD was lower (*P *< 0.04) in Italian participants (19.6%) than in the participants from Kraków (25.3%) and Novosibirsk (32.4%). The clinical and echocardiographic characteristics of the subjects by LVDD group appear in Additional file 1: Tables S9 and S10, respectively.

**Table 5:** Diastolic function grades by study, center and age group

	Diastolic function			
	Normal function	1 group Impaired relaxation	2 group Elevated LV filling pressure	3 group Combined dysfunction
*Study*				
FLEMENGHO	589 (75.3)	73 (9.3)	93 (11.9)	27 (3.5)
EPOGH	380 (79.8)	41 (8.6)	42 (8.8)	13 (2.8)
*Center*				
Gdask	94 (87.0)	7 (6.5)	6 (5.6)	1 (0.9)
Kraków	103 (83.1)	8 (6.4)	10 (8.1)	3 (2.4)
Mirano	85 (80.2)	9 (8.5)	8 (7.5)	4 (3.8)
Novosibirsk	97 (70.4)	18 (13.0)	18 (13.0)	5 (3.6)
*Age group*				
< 30	183 (96.3)	7 (3.7)	--	--
30-39	197 (94.3)	10 (4.8)	2 (0.9)	--
40-49	206 (91.2)	13 (5.7)	4 (1.8)	3 (1.3)
50-59	240 (74.5)	39 (12.1)	34 (10.6)	9 (2.8)
≥ 60	142 (45.7)	45 (14.5)	96 (30.8)	28 (9.0)

Values are number (%). Impaired relaxation (group 1) indicates low E/A and normal E/e'; possible elevated LV filling pressure (group 2) indicates normal E/A and high E/e'; combined dysfunction (group 3) indicates low E/A and high E/e'

### NT-proBNP and LV diastolic dysfunction

**Figure 1:**
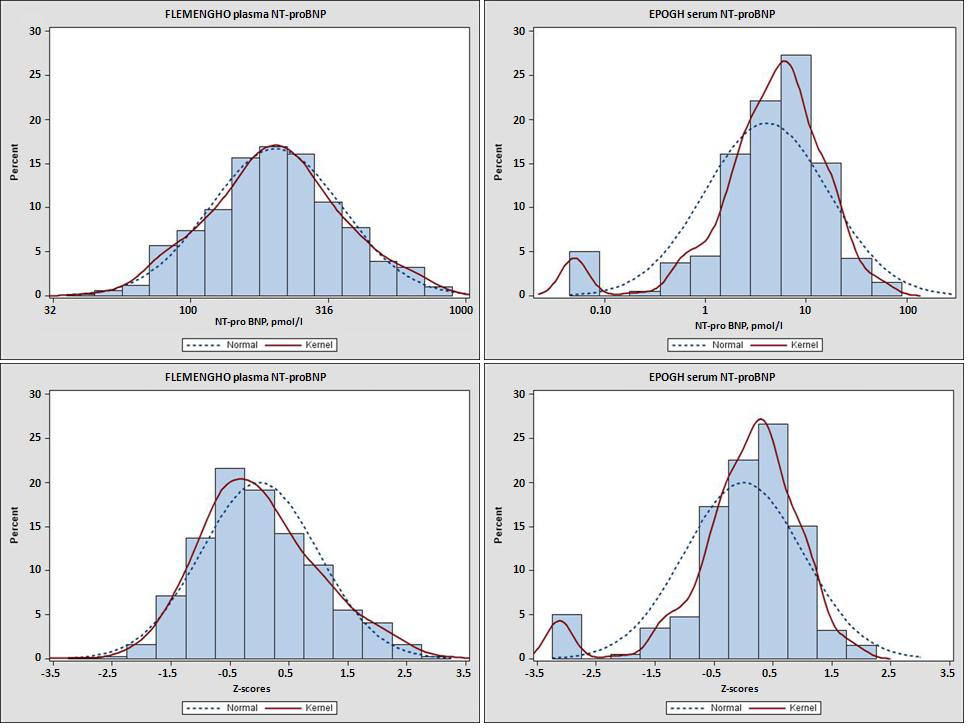
**The histograms of the NT-proBNP levels (A, C) and the corresponding z-scores (B, D) by the FLEMENGHO and EPOGH cohort**.

Figure 1 shows histograms of the logarithmically transformed NT-proBNP levels and the corresponding z-scores by cohort. Compared to the participants with normal diastolic function (n = 969, 77.0%), subjects with elevated LV filling pressure (group 2) had significantly higher NT-proBNP (z-scores, -0.10 ± 0.94 vs. 0.49 ± 1.11; *P *< 0.0001), with a similar trend (-0.10 ± 0.94 vs. 0.21 ± 1.06; *P = *0.007) for those with impaired relaxation (group 1). However, subjects with normal diastolic function and those with combined dysfunction (group 3) had similar levels of NT-proBNP (-0.10 ± 0.94 vs. -0.04 ± 1.11; *P = *0.76).

## Discussion

Epidemiological studies performed in the general population provide an unbiased estimation of the prevalence and prognostic significance of health-related states or events (LVDD in our study). Epidemiology is highly regarded in evidence-based medicine for identifying risk factors for disease and determining optimal treatment approaches for clinical practice. Because the process of myocardial remodeling starts before the onset of symptoms, we place special emphasis on the detection of subclinical (asymptomatic) LV systolic and diastolic dysfunction and the timely identification of patients who are at risk for developing overt HF. Thus, the present report focused on the prevalence and comparison of echocardiographic criteria for LVDD between populations. Mean age of the FLEMENGHO and EPOGH participants was 51.1 and 44.5 years and the prevalence of LVDD was 24.7% and 20.2%. The age-standardized estimated were 22.4% and 25.1%, respectively. The key finding was that age-specific cut-off limits for the transmitral E/A ratio and the threshold for the E/e' ratio, which we used for the classification of LVDD, were consistent and reproducible across two independently recruited population cohorts. The same also applied to the correlates of the E/A, e'/a' and E/e' ratios. These observations lend support to the clinical applicability of the criteria for the classification of LVDD as proposed in this report.

The reported prevalence of LVDD depends on several factors, such as the characteristics of the population under study, imaging techniques, and the criteria applied to diagnose or to grade LVDD (Additional file 1: Table S1). Redfield [2] and Abhayaratna [3] used similar, but not identical pulsed wave Doppler and TDI criteria. Three studies^4-6 ^defined LVDD exclusively based on transmitral E/A ratio cutoffs. Fisher [7] and Azevedo [8] applied different combinations of the criteria proposed by the European Working Group on Diastolic Heart Failure in 1998 [17]. The criteria proposed in the recently published recommendations [18] for the evaluation of LV diastolic function by echocardiography require further testing for their applicability in the general population.

In all aforementioned studies participants were randomly recruited from populations (Additional file 1: Table S2). Age at recruitment was more than 45 years in 3 reports [2,4,8] and more than 55 years in the Rotterdam Study [5]. The Augsburg study [7] encompassed an age range from 25 to 75 years, whereas the Canberra study [3] enrolled only elderly from 60 to 86 years. The prevalence of hypertension and diabetes ranged from 28.2% to 75.0% and from 3.3% to 54.7%, respectively. The prevalence of any grade of LVDD ranged from 11.1% in Augsburg [7] to 34.7% (7.3%) in Canberra [3] (Additional file 1: Table S1). In our current study, the frequency of any grade of LVDD was 23.0%. This estimate has to be interpreted, keeping in mind that age averaged 48.6 years (range, 14.2-89.5 years), and the proportions of women and patients with hypertension or diabetes mellitus were 42.5%, 42.4% and 3.1%, respectively.

Our current study showed that 3.5% and 2.8% of the FLEMENGHO and EPOGH participants had a low E/A ratio in the presence of an elevated E/e' ratio. To our knowledge, this group of patients was not described in previous reports [2,3] with the exception of our previous FLEMENGHO report [9]. In the current report, we confirmed the existence of this type of LVDD in EPOGH participants. The underlying mechanisms require clarification. Parallel increases in the pressures in the LA and LV during diastole might contribute to this type of LVDD.

Recent community-based studies [2,4,19,20] explored the prognostic role of classical Doppler indexes and the new TDI-derived parameters. In the Olmsted study [2] mild LVDD (hazard ratio, 8.31; *P *< 0.001) and moderate or severe diastolic dysfunction (10.2; *P *< 0.001) predicted all-cause mortality, while controlling for age, sex, and EF. However, in this study, the authors did not adjust the models for other important cardiovascular risk factors. In 1036 participants enrolled in the Copenhagen City Heart study [19], low systolic and A' velocities derived from colour Doppler imaging and averaged from 16 myocardial segments independently predicted total mortality [19]. The authors did not explore whether the new TDI indexes captured prognostic information over and beyond classical Doppler measurements of diastolic function as it was shown in previous studies [4,20]. For instance, In the Cardiovascular Health Study,[20] the adjusted risk of development of overt HF was highest at the extremes of the distribution of the transmitral E/A ratio. In the Strong Heart Study,[4] all-cause and cardiac mortality had a U-shaped relation with the E/A ratio.

In the absence of an outcome-driven age-specific diagnostic reference frame, averaging the 2.5 and 97.5 percentiles for the E/A ratio in subjects free from cardiovascular diseases included in the FLEMENGHO and EPOGH cohorts, and rounding the resulting boundaries to the closest integer value, produced working definitions of normal LV diastolic function, which can be easily remembered. Following this approach, absolute values for the normal lower limit of the E/A ratio, would be higher than 1.2, 1.0, 0.9, 0.8, and 0.7 in subjects aged < 30, 30-39, 40-49, 50-59, and > 60 years, respectively. The lower boundaries of the age-specific thresholds for the E/A ratio decreased approximately by 0.10 per decade of age. In the FLEMENGHO and EPOGH studies, the 97.5th percentiles of E/e' ratio in the reference groups were 8.6 and 8.5, respectively. These findings are in line with the data reported from invasive studies, that an E/e' ratio of less than 8 accurately predicts normal LV filling pressure [17,21].

Because NT-proBNP values vary with the degree of LVDD,^23 ^we also compared the levels of NT-proBNP among the LVDD groups. In line with our previously published FLEMENGHO findings,[9] we observed higher levels in subjects with impaired relaxation pattern (group 1) and in subjects with elevated LV filling pressure (group 2). However, in subjects who had an elevated E/e' ratio and an abnormally low age-specific E/A (group 3), the NT-proBNP levels were not different from those in subjects with normal LV function.

Our study has to be interpreted within the context of its potential limitations and strengths. First, the Doppler blood flow measurements and the TDI velocities are prone to measurement error. In the present study, one experienced observer in each center recorded all Doppler images using a common highly standardized imaging protocol [9]. Furthermore, all digitally stored images were centrally post-processed by one observer. Second, because NT-proBNP was measured by different methods in FLEMENGHO and EPOGH, we reported z-scores instead of the measured values of NT-proBNP. However, within cohorts the associations between the classification of LVDD and circulating NT-proBNP levels were consistent. Third, the patterns of transmitral flow and mitral annulus velocities depend to some extent on the compliance and contractile function of the left atrium. Thus, the evaluation of diastolic function using the proposed cut-offs might not be applicable to patients with sustained atrial fibrillation. Fourth, in continuous analyses, the correlates of LVDD in two cohorts were the same and in line with previous reports [3-5]. This observation might be considered as a validation of our current results.

## Conclusions

In conclusion, LVDD has a prevalence of over 20% in European populations. To classify different grades of LVDD, we applied age-specific diagnostic thresholds based on a combination of Doppler velocities ratios derived from transmitral blood flow and mitral annular movement. We demonstrated that these thresholds were consistent and reproducible across population cohorts. This diagnostic classification needs validation in prospective studies in terms of progression of disease and as predictor of cardiovascular complications. Data collection to meet these goals is currently in progress in the FLEMENGHO and EPOGH cohorts.

## Competing interests

The authors declare that they have no competing interests.

## Authors' contributions

JAS is the principal investigator of the European Project on Genes in Hypertension (EPOGH), a large scale epidemiological project that addresses different questions related to genetic and environmental risk factors for cardiovascular diseases. MKB, TK and JAS have made substantial contribution to study conception and design, as well as data acquisition, analysis and interpretation, and drafting of the manuscript. All authors listed in this manuscript substantially contributed to the collection of echocardiographic data, revising critically the draft of the manuscript for important intellectual content and finally approving it.

## Supplementary Material

Additional file 1 **Below is the link to the electronic supplementary material**. Supplementary Tables (DOC 420 kb). Click here for file
