# Supplementary material for: Prevalence of left ventricular diastolic dysfunction in European populations based on cross-validated diagnostic thresholds
Source: Cardiovasc Ultrasound. 2012 Mar 19;10:10. doi: 10.1186/1476-7120-10-10 (PMC3351014; doi:10.1186/1476-7120-10-10)
Supplement: Additional file 1 — Below is the link to the electronic supplementary material. Supplementary Tables (DOC 420 kb). [file 1476-7120-10-10-S1.DOC]

**Data Supplement**

**Prevalence of left ventricular diastolic dysfunction in European populations based on cross-validated diagnostic thresholds**Short title: Population-based criteria for LV diastolic dysfunction

Supplemental Table S1. Factor influencing the prevalence of diastolic dysfunction in several population studies

| **Population Study** | **Any grade of diastolic dysfunction (%)** | **Criteria for classification of diastolic function** | | | | **Not classified (%)** |
| --- | --- | --- | --- | --- | --- | --- |
|  |  | Normal | Mild | Moderate | Severe |  |
| Olmsted County^2^, n=2042 | 28.1 | 0.75<E/A<1.5;  DT>140 ms;  S≥D;  ARdur<Adur; E/e_lat_<10 | E/A≤0.75;  DT―; S>D;  ARdur<Adur; E/e_lat_<10 | 0.75<E/A<1.5;  DT>140 ms;   S<D or  ARdur<Adur+30ms; E/e_lat_≥10; at least 2 Doppler criteria | E/A>1.5;  DT<140 ms;   S<D or  ARdur<Adur+30ms; E/e_lat_≥10; at least 2 Doppler criteria | 12.9 |
| Canberra Cohort^3^, n=1275 | 34.7 | 0.75<E/A<1.5;  DT>160 ms;  S>D;  ARdur<Adur; E/e_lat_<10 | E/A<0.75;  DT>240 ms; S>D;  ARdur<Adur; E/e_lat_<10 | 0.75<E/A<1.5;  DT>160 ms;   S<D or  ARdur<Adur+30ms; E/e_lat_≥10; at least 2 Doppler criteria | E/A>1.5;  DT<160 ms;   S<D or  ARdur<Adur+30ms; E/e_lat_≥10; at least 2 Doppler criteria | 2.5 |
| Strong Heart Study^4^, n=3008 | 19.0 | 0.60<E/A<1.5 | E/A<0.60 | E/A>1.5 | | ― |
| Rotterdam Study^5^, n=4425 | 31.6 | 0.75≤E/A≤1.5 | E/A<0.75 | E/A>1.5 | | 3.8 |
| Jackson Cohort of the ARIC Study^6^, n=1849 | 14.8 | 0.70≤E/A≤1.5 | E/A<0.70 | E/A>1.5 | | ― |
| MONICA Augsburg Study^7^, n=1274 | 11.1 | E/A_<50 years_ ≥1.0, E/A_>50 years_ ≥0.5 or IVRT_<30 years_ ≤92ms, IVRT_30-50 years_ ≤100ms, IVRT_>50 years_ ≤105ms | EF>45% and  E/A_<50 years_ <1.0; E/A_>50 years_ <0.5 or IVRT_<30 years_ >92ms; IVRT_30-50 years_ >100ms IVRT_>50 years_ >105ms | | | ― |
| Porto Population Study^8^, n=684 | 25.0 | E/A_<50 years_ ≥1.0, E/A_≥50 years_ ≥0.5 and DT_<50 years_ ≤220 ms,  DT_≥50 years_ ≤280 ms and/or IVRT_<50 yeas_ ≤100 ms; IVRT_≥50 years_ ≤105 ms | E/A_<50 years_ <1.0, E/A_≥50 years_ <0.5 and DT_<50 years_ >220 ms,  DT_≥50 years_ >280 ms and/or IVRT_<50 yeas_ >100 ms; IVRT_≥50 years_ >105 ms | | | ― |

Supplemental Table S2. Factor influencing the prevalence of diastolic dysfunction in several population studies (continue)

| **Population Study** | **Age range (mean±SD, years)** | **Participation rate (%)** | **Women  (%)** | **HT  (%)** | **Diabetes (%)** |
| --- | --- | --- | --- | --- | --- |
|  | | | |
| Olmsted County^2^, n=2042 | ≥45  (63±11) | 47.0 | 53.0 | 28.2 | 4.5 |
| Canberra Cohort^3^, n=1275 | 60─86 (69±7.0) | 75.2 | 50.0 | 47.2 | 10.3 |
| Strong Heart Study^4^, n=3008 | 45─74 (60±8.0) | 89.0 | 64.0 | 45.9 | 54.7 |
| Rotterdam Study^5^, n=4425 | ≥55 (71±7.5) | 74.9 | 60.8 | NR | 12.6 |
| Jackson Cohort of the ARIC Study^6^, n=1849 | NR (59±5.7) | NR | 65.0 | 60.6 | 23.0 |
| MONICA Augsburg Study^7^, n=1274 | 25─75 (51±11) | 70.6 | 52.7 | 33.7 | 3.3 |
| Porto Population Study^8^, n=684 | ≥45  (61±11) | 70.0 | 60.8 | 75.0* | 16.8 |

*In the Porto Population Study HT was defined as SBP≥130 mmHg and/or DBP≥85 mmHg or antihypertensive drug treatment. NR, not reported.

**Supplemental Table S3.** Characteristics of participants – EPOGH study.

| **Characteristic** | **Gdańsk**  **n=108** | **Kraków  n=124** | **Mirano n=106** | **Novosibirsk n=138** |
| --- | --- | --- | --- | --- |
| *Anthropometrics* |  |  |  |  |
| Female (%) | 48 (44.4) | 67 (54.0) | 64 (60.4)^G^ | 86 (62.3)^G^ |
| Age, years | 40.9 ± 14.4 | 40.6 ± 14.9 | 48.7 ± 14.3^GK^ | 47.5 ± 15.2^GK^ |
| Weight, kg | 80.9 ± 16.9 | 74.1 ± 16.8^G^ | 73.9 ± 15.1^G^ | 76.5 ± 16.1 |
| Height, cm | 170.8 ± 8.33 | 168.3 ± 8.81 | 166.4 ± 8.94^G^ | 166.7 ± 9.2^G^ |
| Body Mass Index, kg/m^2^ | 27.6 ± 4.7 | 26.1 ± 5.26 | 26.7 ± 4.9 | 27.6 ± 5.6^C^ |
| Waist circumference, cm | 92.8 ± 13.8 | 87.8 ± 15.5^G^ | 91.3 ± 13.9 | 87.3 ± 12.5^GM^ |
| Systolic pressure, mm Hg | 128.9 ± 15.9 | 134.3 ± 18.8 | 124.1 ± 16.6^K^ | 133.3 ± 24.2^M^ |
| Diastolic pressure, mm Hg | 76.4 ± 9.3 | 81.7 ± 12.5^G^ | 81.1 ± 10.3^G^ | 84.9 ± 14.6^M^ |
| Heart rate, beats/min | 67.1 ± 10.2 | 66.5 ± 10.3 | 67.2 ± 11.3 | 67.1 ± 10.5 |
| *Questionnaire data* |  |  |  |  |
| Current smoking, n (%) | 27 (27.5) | 22 (17.7) | 20 (18.9) | 33 (23.9) |
| Drinking alcohol, n (%) | 28 (25.9) | 16 (12.9)^G^ | 50 (47.2)^GK^ | 41 (29.7)^CM^ |
| Hypertensive, n (%) | 32 (29.6) | 61 (49.2)^G^ | 48 (45.3)^G^ | 70 (50.7)^G^ |
| Treated for hypertension, n (%) | 13 (13.3) | 38 (30.6)^G^ | 27 (25.5)^G^ | 49 (35.5)^G^ |

Values are mean (±SD), or number of subjects (%). ^G^*P*<0.05 *vs* Gdańsk, ^K^*P*<0.05 *vs* Kraków, ^M^*P*<0.05 *vs* Mirano.

**Supplemental Table S4. Echocardiographic characteristics of participants – EPOGH study.**

| **Characteristic** | **Gdańsk  n=108** | **Kraków  n=124** | **Mirano n=106** | **Novosibirsk n=138** |
| --- | --- | --- | --- | --- |
| *Conventional echocardiography* |  |  |  |  |
| Left atrium, cm | 4.01 ± 0.57 | 3.88 ± 0.58 | 3.89 ±0.51 | 3.94 ± 0.51 |
| LV internal diameter, cm | 5.12 ± 0.41 | 5.06 ±0.45 | 4.86 ± 0.45^GC^ | 4.97 ± 0.39^G^ |
| Interventricular septum, cm | 0.95 ± 0.14 | 0.94 ±0.17 | 0.92 ± 0.15 | 0.97 ± 0.18^M^ |
| Posterior wall, cm | 0.91 ± 0.12 | 0.85 ± 0.14^G^ | 0.86 ± 0.13^G^ | 0.88 ± 0.14^C^ |
| Relative wall thickness | 0.36 ± 0.048 | 0.35 ± 0.057 | 0.37 ± 0.06 | 0.37 ± 0.06^C^ |
| LV mass index, g/m^2^ | 90.5 ± 19.6 | 88.1 ± 21.0 | 82.98 ± 18.9^GC^ | 89.4 ± 21.2^M^ |
| Ejection fraction, % | 64.1 ± 6.52 | 63.5 ± 5.90 | 64.9 ± 7.06 | 64.6 ± 7.13 |
| *Doppler data* |  |  |  |  |
| E peak, m/s | 0.75 ± 0.13 | 0.75 ± 0.17 | 0.70 ± 0.14^GC^ | 0.67 ± 0.14^GC^ |
| A peak, m/s | 0.59 ± 0.14 | 0.55 ± 0.15 | 0.60 ± 0.19 | 0.60 ± 0.18^C^ |
| E/A ratio | 1.35 ± 0.43 | 1.47 ± 0.60 | 1.29 ± 0.48^C^ | 1.23 ± 0.51^C^ |
| e’ peak*, cm/s | 12.2 ± 3.56 | 11.6 ± 3.66 | 10.6 ± 3.29^GC^ | 10.6 ± 3.68^GC^ |
| a’ peak*, cm/s | 9.20 ± 1.87 | 8.59 ± 1.92^G^ | 10.0 ± 2.14^GC^ | 9.24 ± 2.17^CM^ |
| e’/a’ ratio* | 1.46 ± 0.67 | 1.49 ± 0.72 | 1.16 ± 0.56^GC^ | 1.31 ± 0.75^C^ |
| E/e’ ratio | 6.60 ± 1.88 | 6.93 ± 2.14 | 7.07 ± 2.22 | 6.88 ± 2.40 |

Values are mean (±SD). LV, left ventricle. ^G^*P*<0.05 *vs.* Gdańsk, ^C^*P*<0.05 *vs.* Kraków, ^M^*P*<0.05 *vs.* Mirano.

**Supplemental Table S5.** Transmitral E/A, TDI e’a’, left atrial volume index, and averaged E/e’ in the reference groups - FLEMENGHO and EPOGH cohorts combined

| Age | **<30** | **30-39** | **40-49** | **50-59** | **≥60** | **All** | Age | **<30** | **30-39** | **40-49** | **50-59** | **≥60** | **All** |
| --- | --- | --- | --- | --- | --- | --- | --- | --- | --- | --- | --- | --- | --- |
| **E/A ratio** |  |  |  |  |  |  | **LAVI** |  |  |  |  |  |  |
| N | 142 | 131 | 122 | 92 | 36 | **523** | N | 142 | 131 | 122 | 92 | 36 | **523** |
| X | 1.97 | 1.67 | 1.49 | 1.18 | 0.97 | **1.57** | X | 17.6 | 18.4 | 19.9 | 21.6 | 22.0 | **19.3** |
| SD | 0.48 | 0.42 | 0.35 | 0.23 | 0.21 | **0.50** | SD | 3.48 | 3.64 | 4.10 | 4.19 | 4.68 | **4.17** |
| P2.5 | 1.23 | 1.02 | 0.91 | 0.77 | 0.67 | **0.80** | P2.5 | 12.2 | 11.8 | 13.4 | 15.3 | 13.4 | **12.4** |
| P5 | 1.28 | 1.11 | 0.94 | 0.79 | 0.67 | **0.87** | P5 | 12.4 | 12.9 | 13.8 | 15.9 | 14.2 | **13.3** |
| P10 | 1.36 | 1.23 | 1.06 | 0.89 | 0.70 | **0.98** | P10 | 13.0 | 13.6 | 14.4 | 16.7 | 14.4 | **14.1** |
| P25 | 1.65 | 1.34 | 1.25 | 1.02 | 0.82 | **1.23** | P25 | 14.9 | 15.7 | 16.9 | 18.5 | 19.0 | **16.3** |
| P50 | 1.90 | 1.66 | 1.41 | 1.17 | 0.93 | **1.50** | P50 | 17.3 | 18.4 | 19.5 | 20.9 | 22.3 | **19.0** |
| P75 | 2.30 | 1.96 | 1.70 | 1.32 | 1.09 | **1.88** | P75 | 19.9 | 21.0 | 22.7 | 24.9 | 24.7 | **21.9** |
| P90 | 2.51 | 2.11 | 1.96 | 1.51 | 1.28 | **2.21** | P90 | 22.4 | 23.2 | 25.5 | 27.7 | 28.3 | **25.3** |
| P95 | 2.87 | 2.41 | 2.05 | 1.56 | 1.41 | **2.49** | P95 | 24.0 | 25.3 | 27.9 | 28.8 | 29.4 | **27.5** |
| P97.5 | 3.22 | 2.55 | 2.26 | 1.64 | 1.55 | **2.56** | P97.5 | 26.9 | 26.7 | 28.3 | 29.4 | 29.7 | **28.3** |
| **e’/a’ ratio** |  |  |  |  |  |  | **E/e’ ratio** |  |  |  |  |  |  |
| N | 142 | 131 | 122 | 92 | 36 | **523** | N | 142 | 131 | 122 | 92 | 36 | **523** |
| X | 2.37 | 1.81 | 1.51 | 1.11 | 0.87 | **1.70** | X | 5.27 | 5.54 | 5.97 | 6.46 | 7.07 | **5.83** |
| SD | 0.59 | 0.53 | 0.43 | 0.29 | 0.23 | **0.68** | SD | 0.97 | 0.94 | 1.20 | 1.24 | 1.02 | **1.20** |
| P2.5 | 1.41 | 0.94 | 0.85 | 0.59 | 0.38 | **0.66** | P2.5 | 3.74 | 4.09 | 3.83 | 4.49 | 4.38 | **3.86** |
| P5 | 1.57 | 1.03 | 0.94 | 0.64 | 0.54 | **0.77** | P5 | 3.93 | 4.16 | 4.32 | 4.62 | 5.11 | **4.16** |
| P10 | 1.73 | 1.15 | 1.00 | 0.76 | 0.63 | **0.91** | P10 | 4.17 | 4.48 | 4.60 | 5.10 | 6.02 | **4.46** |
| P25 | 1.93 | 1.42 | 1.15 | 0.93 | 0.75 | **1.15** | P25 | 4.62 | 4.87 | 5.09 | 5.49 | 6.40 | **4.97** |
| P50 | 2.31 | 1.77 | 1.46 | 1.10 | 0.83 | **1.64** | P50 | 5.10 | 5.47 | 5.93 | 6.34 | 7.07 | **5.67** |
| P75 | 2.77 | 2.20 | 1.74 | 1.26 | 1.00 | **2.11** | P75 | 5.79 | 6.07 | 6.73 | 7.14 | 7.73 | **6.62** |
| P90 | 3.19 | 2.45 | 2.00 | 1.44 | 1.15 | **2.64** | P90 | 6.70 | 6.73 | 7.53 | 8.18 | 8.67 | **7.52** |
| P95 | 3.43 | 2.68 | 2.30 | 1.68 | 1.43 | **2.95** | P95 | 6.98 | 7.17 | 8.15 | 8.73 | 8.86 | **8.15** |
| P97.5 | 3.89 | 3.00 | 2.54 | 1.78 | 1.46 | **3.22** | P97.5 | 7.66 | 7.47 | 8.85 | 8.84 | 8.99 | **8.60** |

N, X, SD, P_2.5_, P_5_, P_10_, P_25_, P_50_, P_75_, P_90_, P_95_, P_97.5_ indicate number of subjects, mean, standard deviation and percentiles.

**Supplemental Table S6.** Transmitral E/A, TDI e’/a’, left atrial volume index, and averaged E/e’ in the reference group – FLEMENGHO cohort.

| Age | **<30** | **30-39** | **40-49** | **50-59** | **≥60** | **All** | Age | **<30** | **30-39** | **40-49** | **50-59** | **≥60** | **All** |
| --- | --- | --- | --- | --- | --- | --- | --- | --- | --- | --- | --- | --- | --- |
| **E/A ratio** |  |  |  |  |  |  | **LAVI** |  |  |  |  |  |  |
| N | 75 | 54 | 108 | 70 | 31 | **338** | N | 75 | 54 | 108 | 70 | 31 | **338** |
| X | 1.99 | 1.73 | 1.48 | 1.18 | 0.97 | **1.52** | X | 18.0 | 19.0 | 20.0 | 22.0 | 22.0 | **20.0** |
| SD | 0.46 | 0.45 | 0.35 | 0.23 | 0.21 | **0.49** | SD | 3.52 | 3.87 | 4.14 | 4.36 | 4.61 | **4.28** |
| P2.5 | 1.23 | 1.15 | 0.88 | 0.76 | 0.67 | **0.79** | P2.5 | 10.7 | 13.4 | 13.3 | 15.3 | 14.2 | **12.7** |
| P5 | 1.36 | 1.24 | 0.93 | 0.79 | 0.67 | **0.86** | P5 | 12.2 | 13.4 | 13.7 | 16.0 | 14.3 | **13.5** |
| P10 | 1.38 | 1.25 | 1.01 | 0.87 | 0.77 | **0.94** | P10 | 13.1 | 14.2 | 14.5 | 16.7 | 14.4 | **14.5** |
| P25 | 1.66 | 1.43 | 1.25 | 1.05 | 0.83 | **1.19** | P25 | 16.0 | 15.7 | 17.1 | 18.3 | 19.0 | **16.9** |
| P50 | 1.94 | 1.66 | 1.40 | 1.18 | 0.95 | **1.44** | P50 | 18.1 | 18.9 | 19.6 | 21.4 | 21.8 | **19.6** |
| P75 | 2.35 | 1.99 | 1.67 | 1.33 | 1.09 | **1.78** | P75 | 19.9 | 21.7 | 22.7 | 26.0 | 24.7 | **22.7** |
| P90 | 2.50 | 2.14 | 1.96 | 1.51 | 1.25 | **2.17** | P90 | 22.4 | 24.4 | 25.8 | 28.1 | 28.9 | **26.7** |
| P95 | 2.87 | 2.51 | 2.08 | 1.55 | 1.41 | **2.45** | P95 | 24.3 | 26.6 | 27.9 | 29.2 | 29.4 | **28.0** |
| P97.5 | 3.22 | 2.82 | 2.36 | 1.61 | 1.55 | **2.53** | P97.5 | 26.9 | 26.7 | 28.3 | 29.4 | 29.7 | **28.8** |
| **e’/a’ ratio** |  |  |  |  |  |  | **E/e’ Ratio** |  |  |  |  |  |  |
| N | 75 | 54 | 108 | 70 | 31 | **338** | N | 75 | 54 | 108 | 70 | 31 | **338** |
| X | 2.44 | 1.88 | 1.49 | 1.10 | 0.89 | **1.63** | X | 5.17 | 5.57 | 5.99 | 6.45 | 7.11 | **5.93** |
| SD | 0.61 | 0.50 | 0.42 | 0.27 | 0.23 | **0.68** | SD | 0.93 | 0.97 | 1.20 | 1.16 | 1.02 | **1.21** |
| P2.5 | 1.48 | 1.03 | 0.85 | 0.59 | 0.54 | **0.66** | P2.5 | 3.84 | 3.64 | 3.65 | 4.49 | 4.38 | **3.93** |
| P5 | 1.62 | 1.04 | 0.94 | 0.64 | 0.63 | **0.76** | P5 | 3.93 | 4.30 | 4.35 | 4.66 | 5.11 | **4.19** |
| P10 | 1.81 | 1.23 | 1.00 | 0.76 | 0.67 | **0.85** | P10 | 4.15 | 4.38 | 4.60 | 5.17 | 6.13 | **4.46** |
| P25 | 1.93 | 1.57 | 1.15 | 0.92 | 0.77 | **1.10** | P25 | 4.45 | 4.85 | 5.09 | 5.51 | 6.48 | **5.01** |
| P50 | 2.40 | 1.88 | 1.45 | 1.11 | 0.84 | **1.52** | P50 | 5.01 | 5.60 | 5.96 | 6.35 | 7.21 | **5.85** |
| P75 | 2.84 | 2.20 | 1.72 | 1.26 | 1.07 | **1.97** | P75 | 5.75 | 6.11 | 6.73 | 7.13 | 7.78 | **6.73** |
| P90 | 3.30 | 2.56 | 2.00 | 1.39 | 1.15 | **2.57** | P90 | 6.61 | 6.91 | 7.53 | 8.13 | 8.21 | **7.54** |
| P95 | 3.51 | 3.00 | 2.30 | 1.54 | 1.43 | **2.96** | P95 | 6.86 | 7.22 | 8.29 | 8.61 | 8.86 | **8.17** |
| P97.5 | 3.99 | 3.07 | 2.54 | 1.78 | 1.46 | **3.22** | P97.5 | 7.66 | 7.47 | 8.85 | 8.84 | 8.99 | **8.61** |

N, X, SD, P_2.5_, P_5_, P_10_, P_25_, P_50_, P_75_, P_90_, P_95_, P_97.5_ indicate number of subjects, mean, standard deviation and percentiles.

Supplemental Table S7. Transmitral E/A, TDI e’/a’, left atrial volume index, and averaged E/e’ in the reference group - EPOGH cohort.

| Age | **<30** | **30-39** | **40-49** | **50-59** | **≥60** | **All** | Age | **<30** | **30-39** | **40-49** | **50-59** | **≥60** | **All** |
| --- | --- | --- | --- | --- | --- | --- | --- | --- | --- | --- | --- | --- | --- |
| **E/A ratio** |  |  |  |  |  |  | **LAVI** |  |  |  |  |  |  |
| N | 67 | 77 | 14 | 22 | 5 | **185** | N | 67 | 77 | 14 | 22 | 5 | **185** |
| X | 1.93 | 1.63 | 1.54 | 1.18 | 0.87 | **1.66** | X | 17.1 | 18.0 | 18.9 | 20.4 | 21.7 | **18.2** |
| SD | 0.52 | 0.39 | 0.39 | 0.25 | 0.25 | **0.50** | SD | 3.4 | 3.44 | 4.11 | 3.39 | 5.66 | **3.69** |
| P2.5 | 1.23 | 0.98 | 0.95 | 0.78 | 0.67 | **0.83** | P2.5 | 12.4 | 11.0 | 13.9 | 11.5 | 13.4 | **12.3** |
| P5 | 1.23 | 1.01 | 0.95 | 0.89 | 0.67 | **0.94** | P5 | 12.6 | 12.4 | 13.9 | 15.9 | 13.4 | **12.7** |
| P10 | 1.31 | 1.18 | 1.09 | 0.93 | 0.67 | **1.06** | P10 | 13.2 | 13.5 | 14.0 | 16.1 | 13.4 | **13.9** |
| P25 | 1.61 | 1.33 | 1.21 | 0.97 | 0.70 | **1.28** | P25 | 14.8 | 15.5 | 15.3 | 19.1 | 19.4 | **15.3** |
| P50 | 1.83 | 1.65 | 1.64 | 1.11 | 0.83 | **1.67** | P50 | 16.3 | 18.3 | 18.4 | 20.1 | 22.6 | **17.9** |
| P75 | 2.21 | 1.87 | 1.83 | 1.32 | 0.95 | **1.94** | P75 | 19.2 | 20.3 | 22.7 | 22.1 | 24.7 | **20.8** |
| P90 | 2.56 | 2.00 | 2.02 | 1.53 | 1.28 | **2.22** | P90 | 21.8 | 22.1 | 24.4 | 23.7 | 28.3 | **23.3** |
| P95 | 3.09 | 2.39 | 2.05 | 1.66 | 1.28 | **2.25** | P95 | 23.8 | 24.0 | 25.0 | 24.9 | 28.3 | **24.7** |
| P97.5 | 3.25 | 2.55 | 2.05 | 1.72 | 1.28 | **2.93** | P97.5 | 25.4 | 25.4 | 25.0 | 27.7 | 28.3 | **25.4** |
| **e’/a’ ratio** |  |  |  |  |  |  | **E/e’ ratio** |  |  |  |  |  |  |
| N | 67 | 77 | 14 | 22 | 5 | **185** | N | 67 | 77 | 14 | 22 | 5 | **185** |
| X | 2.29 | 1.76 | 1.69 | 1.12 | 0.73 | **1.84** | X | 5.38 | 5.12 | 5.84 | 6.53 | 6.86 | **5.65** |
| SD | 0.56 | 0.55 | 0.48 | 0.34 | 0.25 | **0.66** | SD | 1.01 | 0.92 | 1.30 | 1.49 | 1.10 | **1.13** |
| P2.5 | 1.30 | 0.83 | 0.87 | 0.50 | 0.38 | **0.65** | P2.5 | 3.66 | 4.09 | 3.83 | 4.26 | 6.02 | **3.75** |
| P5 | 1.42 | 0.95 | 0.87 | 0.64 | 0.38 | **0.88** | P5 | 3.75 | 4.16 | 3.83 | 4.62 | 6.02 | **4.11** |
| P10 | 1.59 | 1.13 | 0.98 | 0.65 | 0.38 | **1.02** | P10 | 4.17 | 4.48 | 3.86 | 4.71 | 6.02 | **4.46** |
| P25 | 1.91 | 1.28 | 1.45 | 0.94 | 0.63 | **1.30** | P25 | 4.66 | 4.97 | 5.19 | 5.28 | 6.09 | **4.79** |
| P50 | 2.17 | 1.70 | 1.74 | 1.09 | 0.73 | **1.85** | P50 | 5.20 | 5.42 | 5.79 | 6.33 | 6.57 | **5.40** |
| P75 | 2.66 | 2.19 | 1.97 | 1.26 | 0.90 | **2.33** | P75 | 6.07 | 6.05 | 6.46 | 7.95 | 6.92 | **6.27** |
| P90 | 3.08 | 2.45 | 2.27 | 1.46 | 1.02 | **2.70** | P90 | 6.86 | 6.67 | 8.05 | 8.52 | 8.73 | **6.94** |
| P95 | 3.25 | 2.68 | 2.57 | 1.75 | 1.02 | **2.95** | P95 | 7.28 | 6.94 | 8.15 | 8.73 | 8.73 | **8.05** |
| P97.5 | 3.61 | 2.85 | 2.57 | 1.98 | 1.02 | **3.22** | P97.5 | 7.80 | 8.24 | 8.15 | 8.63 | 8.73 | **8.52** |

N, X, SD, P_2.5_, P_5_, P_10_, P_25_, P_50_, P_75_, P_90_, P_95_, P_97.5_ indicate number of subjects, mean, standard deviation and percentiles.

**Supplemental Table S8.** Transmitral E and A velocities and mitral annulus TDI e’ and a’ velocities in the healthy reference groups - FLEMENGHO and EPOGH cohorts combined

| Age | **<30** | **30-39** | **40-49** | **50-59** | **≥60** | **All** | Age | **<30** | | | **30-39** | | **40-49** | | **50-59** | | **≥60** | | **All** |
| --- | --- | --- | --- | --- | --- | --- | --- | --- | --- | --- | --- | --- | --- | --- | --- | --- | --- | --- | --- |
| **Transmitral E, cm/s** | |  |  |  |  |  | **Mitral annulus e’, cm/s** | | | |  | |  | |  | |  | |  |
| N | 142 | 131 | 122 | 92 | 36 | **523** | N | | 142 | 131 | | 122 | | 92 | | 36 | | **523** | |
| X | 84.8 | 78.5 | 79.6 | 72.0 | 67.2 | **78.6** | X | | 16.3 | 14.3 | | 13.5 | | 11.3 | | 9.6 | | **13.8** | |
| SD | 13.9 | 13.9 | 14.5 | 13.5 | 10.4 | **14.7** | SD | | 2.36 | 2.10 | | 2.12 | | 2.11 | | 1.17 | | **2.90** | |
| P2.5 | 58.7 | 51.4 | 54.0 | 46.5 | 47.6 | **49.5** | P2.5 | | 12.2 | 10.2 | | 9.6 | | 7.3 | | 7.1 | | **8.5** | |
| P5 | 65.2 | 55.1 | 55.9 | 48.2 | 49.5 | **54.0** | P5 | | 13.0 | 10.7 | | 10.1 | | 7.8 | | 7.3 | | **9.0** | |
| P10 | 67.1 | 61.2 | 61.5 | 52.4 | 50.9 | **60.0** | P10 | | 13.6 | 11.4 | | 11.1 | | 8.5 | | 8.1 | | **9.9** | |
| P25 | 75.1 | 69.6 | 69.3 | 62.3 | 59.5 | **68.5** | P25 | | 14.7 | 12.9 | | 11.9 | | 9.9 | | 8.8 | | **11.6** | |
| P50 | 85.0 | 78.8 | 80.6 | 70.5 | 67.1 | **78.8** | P50 | | 16.2 | 14.6 | | 13.7 | | 11.3 | | 9.6 | | **14.0** | |
| P75 | 93.9 | 87.4 | 88.2 | 82.2 | 75.3 | **88.1** | P75 | | 17.8 | 15.9 | | 15.2 | | 12.7 | | 10.4 | | **15.8** | |
| P90 | 103 | 95.8 | 98.9 | 91.3 | 80.2 | **96.2** | P90 | | 19.5 | 16.8 | | 16.1 | | 14.1 | | 10.8 | | **17.5** | |
| P95 | 108 | 99.3 | 103 | 94.7 | 84.5 | **103** | P95 | | 20.4 | 17.5 | | 17.1 | | 14.8 | | 11.0 | | **18.6** | |
| P97.5 | 114 | 107 | 107 | 96.9 | 90.2 | **108** | P97.5 | | 21.2 | 18.0 | | 17.4 | | 15.6 | | 13.1 | | **19.6** | |
|  |  |  |  |  |  |  |  | |  |  | |  | |  | |  | |  | |
|  |  |  |  |  |  |  |  | |  |  | |  | |  | |  | |  | |
| **Transmitral A, cm/s** | | |  |  |  |  | **Mitral annulus a’, cm/s** | | | | |  | |  | |  | |  | |
| N | 142 | 131 | 122 | 92 | 36 | **523** | N | | 142 | 131 | | 122 | | 92 | | 36 | | **523** | |
| X | 44.7 | 48.7 | 55.2 | 62.2 | 70.7 | **53.0** | X | | 7.24 | 8.46 | | 9.48 | | 10.6 | | 11.3 | | **8.94** | |
| SD | 9.0 | 10.4 | 10.3 | 11.2 | 12.6 | **12.9** | SD | | 1.44 | 1.82 | | 1.77 | | 1.64 | | 1.88 | | **2.15** | |
| P2.5 | 28.1 | 30.2 | 36.4 | 39.8 | 47.8 | **31.1** | P2.5 | | 4.57 | 5.30 | | 6.16 | | 7.67 | | 7.56 | | **5.20** | |
| P5 | 31.1 | 32.4 | 38.1 | 45.5 | 51.8 | **33.4** | P5 | | 4.97 | 5.68 | | 6.75 | | 8.25 | | 7.69 | | **5.68** | |
| P10 | 35.0 | 34.9 | 42.0 | 50.9 | 54.1 | **37.5** | P10 | | 5.44 | 6.37 | | 7.46 | | 8.81 | | 8.81 | | **6.31** | |
| P25 | 38.2 | 42.3 | 47.9 | 54.9 | 61.9 | **43.2** | P25 | | 6.18 | 7.15 | | 8.35 | | 9.40 | | 9.86 | | **7.37** | |
| P50 | 43.7 | 48.2 | 56.1 | 61.7 | 69.3 | **51.7** | P50 | | 7.16 | 8.22 | | 9.37 | | 10.5 | | 11.3 | | **8.76** | |
| P75 | 50.9 | 54.1 | 63.4 | 68.7 | 79.0 | **61.8** | P75 | | 7.96 | 9.73 | | 10.8 | | 11.8 | | 12.9 | | **10.5** | |
| P90 | 55.5 | 61.9 | 67.9 | 78.0 | 88.1 | **70.0** | P90 | | 9.05 | 11.3 | | 11.8 | | 12.6 | | 13.6 | | **11.9** | |
| P95 | 60.6 | 69.6 | 70.1 | 83.3 | 97.7 | **77.0** | P95 | | 9.79 | 11.7 | | 12.4 | | 13.7 | | 14.1 | | **12.6** | |
| P97.5 | 63.4 | 71.9 | 71.6 | 84.0 | 99.7 | **80.5** | P97.5 | | 10.2 | 12.1 | | 12.9 | | 13.9 | | 15.1 | | **13.4** | |

N, X, SD, P_2.5_, P_5_, P_10_, P_25_, P_50_, P_75_, P_90_, P_95_, P_97.5_ indicate number of subjects, mean, standard deviation and percentiles.

Table S9. Clinical characteristics of participants by diastolic function group – FLEMENGHO and EPOGH cohorts combined

| **Characteristic** | **Normal  function  (n=968)** | **Impaired  relaxation   (n=114)** | **Elevated LV filling pressure  (n=135)** | **Combined dysfunction  (n=40)** |
| --- | --- | --- | --- | --- |
| Transmitral E/A ratio | NL | ↓ | NL | ↓ |
| E/E’ ratio | NL | NL | ↑ | ↑ |
| Age, y | 44.5 ± 14.3 | 57.1 ± 14.3* | 65.1 ± 9.4*† | 65.9 ± 9.8*† |
| Women, n (%) | 106 (52.2) | 51 (44.7) | 82 (60.7)† | 27 (67.5)*† |
| Body mass index, kg/m^2^ | 25.9 ± 4.4 | 28.6 ± 4.2* | 29.4 ± 5.2* | 29.7 ± 3.4* |
| Systolic pressure, mm Hg | 125.8±16.1 | 135.8 ± 16.4* | 148.4 ± 20.3*† | 149.1 ± 18.8* |
| Diastolic pressure, mm Hg | 79.1±10.0 | 84.6 ± 10.7* | 82.8 ± 13.2* | 86.8 ± 11.2* |
| Heart rate, beats/minute | 62.6±9.8 | 70.0 ± 12.9* | 60.2 ± 9.8† | 66.3 ± 9.6 |
| *Questionnaire data* |  |  |  |  |
| Current smoking, n (%) | 219 (22.8) | 30 (26.6) | 14 (10.4)*† | 5 (12.5) |
| Drinking alcohol, n (%) | 628 (65.4) | 71 (62.8) | 75 (55.6)* | 22 (55.0) |
| Hypertensive, n (%) | 307 (31.7) | 77 (67.5)* | 115 (85.2)*† | 35 (87.5)*† |
| Treated for hypertension, n (%) | 168 (17.5) | 51 (45.1)* | 82 (60.7)*† | 24 (60.0)* |
| Beta-blockers, n (%) | 89 (9.2) | 25 (22.1)* | 52 (38.5)*† | 15 (37.5)* |
| ACE or ARB, n (%) | 76 (7.8) | 26 (22.8)* | 31 (23.0)* | 13 (32.5)* |
| Diuretics or CCB, n (%) | 98 (10.1) | 26 (22.8)* | 51 (37.8)*† | 17 (42.5)*† |
| History of MI or coronary revascularization, n (%) | 8 (0.83) | 4 (3.5) | 12 (8.9)*† | 6 (15.0)*† |
| Diabetes, n (%) | 24 (2.5) | 11 (9.7)* | 13 (9.6)* | 6 (15.0)* |
| *NT-proBNP, pmol/l* |  |  |  |  |
| FLEMENGHO | 219  (92 to 360) | 273*  (116 to 467) | 322*  (145 to 606) | 253  (78 to 493) |
| EPOGH | 7.07  (0.71 to 16.4) | 9.54  (1.40 to 27.5) | 12.4*  (0.73 to 23.6) | 5.85‡  (0.05 to 9.79) |

Values are mean (±SD), number of subjects (%). NL indicates normal limits; ACE indicates angiotensin-converting enzyme; ARB indicates angiotensin receptor blockers; MI indicates myocardial infarction. Significance for between-groups differences: *p≤0.05 *vs.* normal; † p≤0.05 *vs.* impaired relaxation group; ‡ p≤0.05 *vs.* elevated LV filling pressure group.

Table S10. Echocardiographic characteristics of participants by diastolic function group – FLEMENGHO and EPOGH cohorts combined

| **Characteristic** | | **Normal  function  (n=968)** | **Impaired  relaxation   (n=114)** | **Elevated LV filling pressure  (n=135)** | | **Combined dysfunction  (n=40)** |
| --- | --- | --- | --- | --- | --- | --- |
| Transmitral E/A ratio | | NL | ↓ | NL | ↓ | |
| E/E’ ratio | | NL | NL | ↑ | ↑ | |
| Conventional echocardiography | | | | | | |
| Left atrium volume index | | 21.2 ± 5.5 | 22.8 ± 6.0* | 28.1 ± 6.9* | 26.5 ± 6.6* | |
| LV internal diameter, cm | | 5.02 ± 0.44 | 5.05 ± 0.53 | 5.05 ± 0.58 | 5.04 ± 0.46 | |
| Interventricular septum, cm | | 0.94 ± 0.15 | 1.04 ± 0.16* | 1.09 ± 0.16*† | 1.17 ± 0.19† | |
| Posterior wall, cm | | 0.86 ± 0.13 | 0.94 ± 0.12* | 0.98 ± 0.13*† | 1.04 ± 0.14* | |
| Relative wall thickness | | 0.36 ± 0.06 | 0.39 ± 0.07* | 0.41 ± 0.07* | 0.44 ± 0.07* | |
| LV mass index, g/m^2^ | | 86.8 ± 18.9 | 97.3 ± 21.3* | 106.8 ± 24.7*† | 115.1 ± 25.7* | |
| Ejection fraction, % | | 65.8 ± 6.9 | 65.2 ± 8.6 | 68.8 ± 8.6* | 65.2 ± 8.6 | |
|  | |  |  |  |  | |
|  | |  |  |  |  | |
| Ejection fraction < 50%, n (%) | | 2 (0.21) | 4 (3.5)* | 3 (2.2)* | 2 (5.0)* | |
| Transmitral Doppler data | | | | | | |
| E peak, cm/s | 76.3 ± 14.6 | | 54.3 ± 10.5* | 78.1 ± 13.9† | 59.2 ± 13.8*‡ | |
| A peak, cm/s | 57.4 ± 14.2 | | 75.9 ± 13.5* | 80.2 ± 15.9* | 90.7 ± 18.6* | |
| E/A ratio | 1.42 ± 0.47 | | 0.73 ± 0.16* | 1.00 ± 0.23*† | 0.66 ± 0.09*‡ | |
| Tissue Doppler velocities**#** | | | | | | |
| e’ peak, cm/s | | 12.5 ± 3.22 | 8.49 ± 2.10* | 7.34 ± 1.29*† | 5.44 ± 1.20*†‡ | |
| a’ peak, cm/s | | 9.42 ± 2.08 | 11.7 ± 1.87* | 10.3 ± 1.78*† | 11.2 ± 1.29* | |
| e’/a’ ratio | | 1.46 ± 0.66 | 0.76 ± 0.28* | 0.75 ± 0.21*† | 0.49 ± 0.10*†‡ | |
| E/e’ ratio | | 6.35 ± 1.40 | 6.58 ± 1.27 | 10.8 ± 1.98*† | 11.1 ± 2.53*† | |

Values are mean (±SD). NL indicates normal limits; LV indicates left ventricle. Significance for between-groups differences: **P*≤0.05 *vs.* normal; † *P*≤0.05 *vs.* impaired relaxation group; ‡ *P*≤0.05 *vs.* elevated LV filling pressure group. # Averaged of septum, lateral, inferior and posterior mitral annulus sites.

**Appendix**

The following investigators participated in the EPOGH Study:

*Belgium* (Leuven) –R. Fagard, L. Thijs, Y. Jin, T. Kuznetsova, T. Richart, and J.A. Staessen;

*Czech Republic* (Pilsen and Prague) – O. Beran, J. Filipovský, L. Golán, T. Grus, G. Grusová, M. Jachymová, J. Seidlerová, Z. Marecková, J. Peleška, V. Svobodova, and M. Dolejsová;

*Germany* (Münster) – E. Brand, and S.M. Brand;

*Italy* (Padua) – E. Casiglia and V. Tikhonoff;

*Poland* (Kraków) – M. Cwynar, J. Gąsowski, T. Grodzicki, K. Kawecka-Jaszcz, M. Kloch-Badełek, M. Loster, A. Olszanecka, A. Sałakowski, K. Stolarz-Skrzypek, B. Wizner, and W. Wojciechowska; (Gdansk) – K. Kunicka, K. Narkiewicz, W. Sakiewicz, E. Swierblewska, and M. Wójtowicz;

*Romania* (Bucharest) – S. Babeanu, D. Jianu, C. Sandu, D. State, and M. Udrea;

*Russian Federation* (Novosibirsk) – T. Kuznetsova, S. Malyutina, Y. Nikitin, E. Pello, A. Ryabikov, and M. Voevoda.

*Project Coordinator* – J.A. Staessen;

*Scientific Coordinator* – K. Kawecka-Jaszcz;

*Steering Committee* – E. Casiglia, J. Filipovský , K. Kawecka-Jaszcz, Y. Nikitin, and J.A. Staessen;

*Data Management Committee* – T. Kuznetsova, J.A. Staessen, K. Stolarz-Skrzypek, and V. Tikhonoff;

*Advisory Committee on Molecular Biology* – G. Bianchi (Universita Vita Salute, Milan, Italy), E. Brand, S.M. Brand, and H.A. Struijker-Boudier [Cardiovascular Research Institute Maastricht (CARIM), Maastricht, The Netherlands].
